# Functional Assessment of Long-Term Microvascular Cardiac Allograft Vasculopathy

**DOI:** 10.3390/jpm13121686

**Published:** 2023-12-05

**Authors:** Noemi Bora, Orsolya Balogh, Tamás Ferenci, Zsolt Piroth

**Affiliations:** 1 Gottsegen National Cardiovascular Center, 1096 Budapest, Hungary; noemi.bora@gokvi.hu (N.B.); orsolya.balogh@gokvi.hu (O.B.); 2 Károly Rácz Doctoral School of Clinical Medicine, Semmelweis University, 1085 Budapest, Hungary; 3 Physiological Controls Group, John von Neumann Faculty of Informatics, Obuda University, 1034 Budapest, Hungary; ferenci.tamas@nik.uni-obuda.hu

**Keywords:** heart transplantation, cardiac allograft vasculopathy, coronary flow reserve, index of microcirculatory resistance

## Abstract

Background: Cardiac allograft vasculopathy (CAV) is a leading cause of death and retransplantation following heart transplantation (HTX). Surveillance angiography performed yearly is indicated for the early detection of the disease, but it remains of limited sensitivity. Methods: We performed bolus thermodilution-based coronary flow reserve (CFR) and index of microcirculatory resistance (IMR) and fractional flow reserve (FFR) measurements in HTX patients undergoing yearly surveillance coronary angiography without overt CAV. Results: In total, 27 HTX patients were included who had 52 CFR, IMR, and FFR measurements at a mean of 43 months after HTX. Only five measurements were performed in the first year. CFR decreased significantly by 0.13 every year (*p* = 0.04) and IMR tended to increase by 0.98 every year (*p* = 0.051), whereas FFR did not change (*p* = 0.161) and remained well above 0.80 over time. After one year, CFR decreased significantly (*p* = 0.022) and IMR increased significantly (*p* = 0.015), whereas FFR remained unchanged (*p* = 0.72). Conclusions: The functional status of the epicardial coronary arteries of transplanted hearts did not deteriorate over time. On the contrary, a significant decrease in CFR was noted. In view of the increasing IMR, this is caused by the deterioration of the function of microvasculature. CFR and IMR measurements may provide an early opportunity to diagnose CAV.

## 1. Introduction

Heart transplantation (HTX) is the ultimate therapy for patients with end-stage heart failure. Short-term survival after HTX has improved considerably, presently exceeding 85% in one year. On the contrary, despite advances in immunosuppression, surgical techniques, and postoperative patient care, mortality beyond 1 year after HTX has not changed, with a median survival of 14.8 years [1].

Cardiac allograft vasculopathy (CAV) remains the leading long-term cause of death and re-transplantation following HTX. According to the most recent International Society for Heart and Lung Transplantation (ISHLT) registry, CAV is responsible for 32% of patient mortality at 5–10 years. Its incidence increases over time, developing in ∼30% of patients at 5 years and almost 50% at 10 years [2,3,4].

CAV is primarily an immunologically mediated condition with immune and non-immune risk factors, the former being associated with the host’s allogeneic response, likely outweighing the latter. Cytomegalovirus (CMV) infection, the risk of which is increased in all solid organ transplant recipients, also plays an important role in the pathomechanism [5,6]. Acute rejection as well as pre-formed or de novo post-transplant donor-specific alloantibodies (DSAs), especially those targeting human leucocyte antigen (HLA) class II antigens, are key factors in the development of CAV [7,8,9].

Given that HTX results in denervated hearts, post-HTX patients experience no ischemic pain. Consequently, CAV is usually asymptomatic or is only heralded by non-specific symptoms such as fatigue, nausea, or abdominal discomfort. It may eventually result in graft dysfunction and heart failure, or can cause silent myocardial infarctions, severe arrhythmias, or sudden cardiac death.

If CAV is detected early, therapy can be modified in time, and the organ’s lifespan may be prolonged. The latest ISHLT guidelines advocate annual coronary angiography to assess the presence of vasculopathy [10], but this only provides information on large epicardial vessels. Intravascular ultrasound (IVUS) offers more detailed morphological information but is seldom applied and provides no data on the anatomy or function of the microvasculature.

Functional studies are not yet routinely used [11] but may detect microvascular dysfunction and CAV potentially at an earlier stage. Fearon et al. [12] reported on the early changes in coronary physiology in HTX patients, but little is known about the long-term alterations of epicardial and microvascular coronary physiology in these subjects.

Therefore, we sought to characterize the long-term changes in epicardial and microvascular physiology in HTX patients without overt CAV as diagnosed by invasive coronary angiography.

## 2. Materials and Methods

Subjects included in the study were HTX recipients who underwent a routine regular yearly check-up at the Gottsegen National Cardiovascular Center, Budapest, Hungary. During these check-ups, endomyocardial biopsy and coronary angiography were performed, followed by coronary physiology measurements. All patients received induction therapy with antithymocyte globulin followed by standard immunosuppressive therapy, including a calcineurin inhibitor (tacrolimus or cyclosporine), an antiproliferative agent (mycophenolate mofetil), and corticosteroids tapered during the first year at the discretion of the treating cardiologist. In the case of graft rejection, intravenous corticosteroids were used in accordance with the guidelines. The therapeutic serum levels of immunosuppressive agents were regularly monitored and titrated accordingly. Patients also received aspirin (ASA), beta blockers, ACE inhibitors (ACEIs), statins, and calcium channel blockers (CA) as per the discretion of the attending cardiologist. Patients provided their written informed consent.

We included patients after successful HTX who had coronary angiography, endomyocardial biopsy, and invasive functional coronary physiology measurements performed by a single operator (ZP). Patients with significant epicardial coronary artery disease found at the first examination were not included since it could not be determined with certainty if it was CAV or coronary artery disease of the donor heart. Patients with any clinical suspicion of acute rejection, graft dysfunction, or CAV at the first assessment were also excluded. No other exclusion criteria were applied.

In our study, bolus thermodilution was applied to calculate the coronary flow reserve (CFR) and index of microcirculatory resistance (IMR) using PressureWire X (Abbott, Santa Clara, CA, USA), which allowed for a measurement of the fractional flow reserve (FFR) to be taken as well.

**Figure 1:**
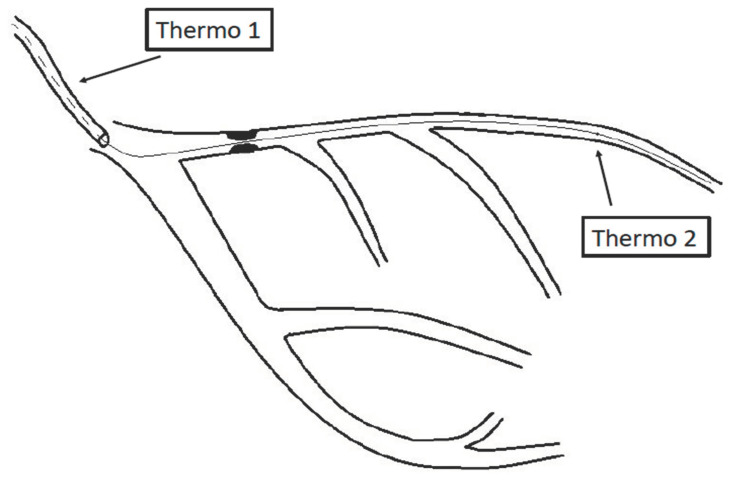
Schematic representation of the bolus thermodilution technique. Thermo 1 denotes the proximal thermistor that records the change of temperature caused by the bolus entering the coronary artery from the guiding catheter, whereas Thermo 2 is the distal thermistor that records the change of the temperature of blood as a consequence of its complete mixing with the bolus.

During coronary angiography, PressureWire X was used to record changes in temperature. PressureWire X was passed to the distal third of the left anterior descending coronary artery (LAD), allowing for the simultaneous measurement of pressure and temperature. The sensor of PressureWire X and the shaft served as the sites of the temperature measurement [13] (Figure 1).

FFR was measured as per standard practice, where P_a_ was the mean aortic pressure and P_d_ was the mean distal coronary pressure measured at maximal hyperemia. Hyperemia was induced by intravenous adenosine administered via the central line (established to perform endomyocardial biopsy) at the standard dose of 140 µg/kg/min. FFR was then calculated as follows:

$$FFR=\frac{P_{d}}{P_{a}}$$

### 2.1. Bolus Thermodilution

When a bolus of saline at a temperature lower than that of blood is briskly injected through the guiding catheter, a V-shaped, time-based temperature change will be recorded in the distal part of the artery. Usually, there is a fast descending and a slower ascending limb [14].

CFR is measured by injecting 3 mL of saline into the coronary artery three times both in resting and in hyperemic states. CFR is the ratio of hyperemic and basal flow. Since the flow and transit time are inversely proportional, CFR can be calculated from the mean transit time at rest (T_mn,rest_) and the hyperemic mean transit time (T_mn,hyper_) as follows [14]:

$$CFR=\frac{T_{mn,rest}}{T_{mn,hyper}}$$

Currently, the most commonly used cut-off value is 2.0, and that has been validated in populations of heart transplant recipients. [15].

### 2.2. IMR

The concept of IMR was first introduced in 2003 in a study by Fearon et al. [16]. During the measurement of IMR, a proxy of minimal microcirculatory resistance, maximal hyperemia is achieved by injecting intracoronary papaverine or by intravenous adenosine infusion, like in CFR measurements. The hyperemic transit time is measured by the injection of 3 mL of room-temperature saline three times during hyperemia and averaging these. Assuming there is a negligible venous pressure compared to the distal coronary pressure measured by the PressureWire, the resistance of the microcirculation (IMR) can be calculated as follows:

$$IMR=\frac{P_{d}}{\frac{1}{T_{mn,hyper}}}$$

IMR is a dimensionless index in close correlation with the actual resistance in the microcirculation, as demonstrated by open-chest swine models by Fearon et al. [16]. There is no universally accepted cut-off value of IMR: in chronic coronary syndrome, an IMR >25 is considered abnormal, whereas in acute ST-segment elevation myocardial infarction, an IMR >40 measured in the culprit vessel immediately after primary percutaneous coronary intervention was found to portend worse prognosis.

In our study, intravenous adenosine was used in the standard dose of 140 µg/kg/min to measure IMR. Central venous pressure was not measured and was considered to be 0.

### 2.3. Statistical Methods

Continuous variables are presented as mean +/− standard deviation and categorical variables are presented as count (percentage). To account for the repeated measurements coming from the same subject (i.e., intra-individual correlation), mixed-effects regression was used to model the time trends, with the outcome being the measured variable—CFR, IMR or FFR—and the predictor variable was either time (assumed to have a linear effect) or, in a later model, time, CMV infection, diabetes mellitus, hypertension, hyperlipidemia, and age (without interaction). A random intercept was added per subject. The model was estimated using restricted maximum likelihood [17]. *p*-values and confidence intervals for the parameters were computed using Satterthwaite denominator degrees of freedom [18]. For the predictions, population-level (no random effects) confidence intervals were calculated with parametric bootstrap using 1000 replicates with the quantile method. Calculations were carried out under the R statistical program package version 4.3.1 [R Core Team (2023)].

## 3. Results

Overall, 27 patients were included in the present analysis who underwent orthotopic heart transplantation between 2002 and 2017. In these subjects, 52 physiological measurements were performed between 2007 and 2018. In total, 17 patients had one, 3 had two, 2 had three, 3 had four, 1 had five, and 1 had six measurements. At least 12 months elapsed between two measurements. Of note, neither AV block nor any other complication were caused in any of the measurements. The mean elapsed time between transplantation and the first measurement was 43 months; only five measurements were performed within 12 months, the vast majority after one year post-HTX. Patient characteristics are summarized in Table 1. The mean donor age was 42.5 ± 8 years, while the average age of the recipients at HTX was 54 ± 12 years. The mean cold ischemic time at HTX was 179 ± 64 min. The majority of the patient population was male with a high prevalence of classical atherosclerotic risk factors. Half of them had undergone HTX for ischemic heart failure. Echocardiography was routinely performed at every measurement. All patients had good graft function, the mean ejection fraction value during the first IMR, CFR, and FFR measurement was 64 ± 10.8%, while right ventricular systolic pressure (RVSP), as estimated by the velocity of the tricuspid regurgitation, was 40 ± 17 mmHg. During follow-up, ejection fraction and RVSP values did not show statistically significant changes, and no evidence of late graft dysfunction was detected among those with repeated measurements.

During follow-up, 85% of patients had at least one episode of—mild to moderate—cellular rejection based on the endomyocardial biopsy results. In total, 81.9% was grade I, while 18.1% was grade II. Grade III rejection was not detected in any of the samples. Humoral rejection was not diagnosed in any of the samples, but testing for humoral rejection was not performed before 2015. Patients with grade I rejection were closely followed without a change in medication, whereas all subjects with grade II acute allograft rejection events were treated by the intensification of immunosuppressive therapy, resulting in the resolution of the rejection as confirmed by follow-up endomyocardial biopsy. DSA was found in only one patient at the time of measurement. During follow-up, seven patients were lost: four died of cancer, two passed away due to acute respiratory distress syndrome caused by bacterial infections, while one died due to graft rejection. No patients underwent heart retransplantation.

CFR measured at the first investigation averaged 3.7 ± 1.79, with an average baseline mean transit time of 0.99 ± 0.41 s and average hyperemic mean transit time of 0.3 ± 0.17 s. IMR at first measurement averaged 23.49 ± 12.79, while mean FFR at first measurement was 0.89 ± 0.05.

**Figure 2:**
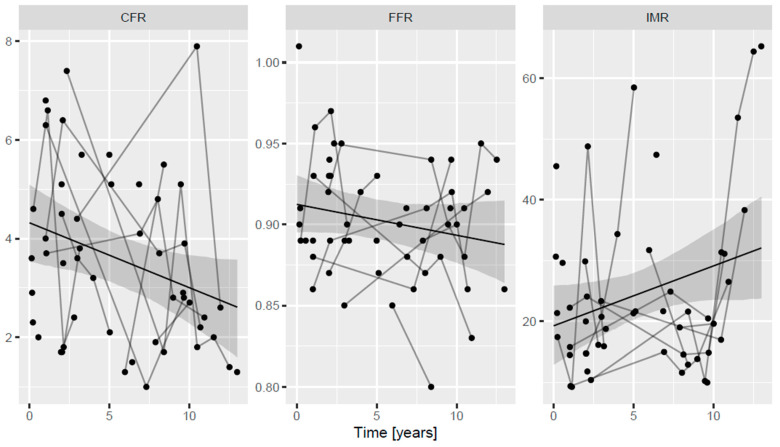
Long-term changes of CFR, IMR and FFR after heart transplantation. Lines connect dots (single measurements) performed in the same individual at different time intervals. CFR coronary flow reserve, FFR fractional flow reserve, IMR index of microcirculatory resistance.

During follow-up, CFR values decreased significantly; when extrapolating our measured values as a function of time, a mean CFR of 4.3 (95% confidence interval (CI), 3.5–5.1) was calculated around the time of HTX, and this decreased by 0.13 (95% CI, 0.01–0.25) every year, a change that reached statistical significance (*p* = 0.04). The calculated extrapolated IMR values at the time of HTX averaged 19.2 (95% CI, 12.5–25.9) and increased by 0.98 (95% CI, 0.00–2.0) every year; this tendency almost reached statistical significance (*p* = 0.051). FFR during follow-up did not change (*p* = 0.161) and remained well above 0.80. The mean calculated extrapolated FFR at the time of HTX was 0.91 (95% CI, 0.89–0.93) with a yearly change of 0.00 (95% CI, 0.00–0.00). This is shown in Figure 2.

In the PITA II Study, Fearon et al. [12] found that microvascular function improved, whereas epicardial physiology worsened in the first year following HTX. Therefore, as a sensitivity analysis, we investigated the effect of elapsed time on the measured physiology parameters, CFR, IMR, and FFR, after 12 months, excluding those five measurements performed during the first year. We found that both CFR (*p* = 0.022) and IMR (*p* = 0.015) showed significant changes: CFR decreased, and IMR increased after 12 months, whereas FFR remained unchanged (*p* = 0.72).

**Figure 3:**
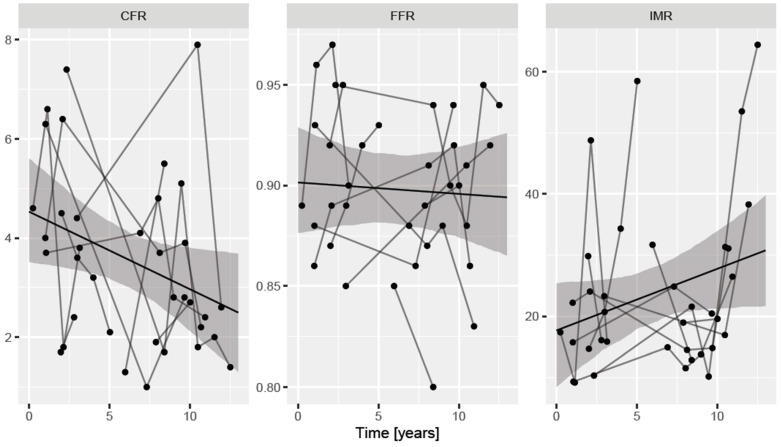
Long-term changes of CFR, IMR and FFR after heart transplantation in patients with repeated physiology measurements. Lines connect dots (single measurements) performed in the same individual at different time intervals. CFR coronary flow reserve, FFR fractional flow reserve, IMR index of microcirculatory resistance.

We also performed a second sensitivity analysis including only those patients (n = 10) who had more than one measurement in our series. In these subjects, a total of 35 measurements were performed. Based on these assessments, CFR decreased significantly by 0.16 every year (*p* = 0.043) and IMR had a non-significant trend to increase by 1.00 every year (*p* = 0.098), whereas FFR showed no change over time (*p* = 0.741). By extrapolation, using these figures, the calculated CFR and IMR at the time of HTX in this subgroup were 4.5 (95% CI, 3.4–5.6) and 17.7 (95% CI, 8.8–26.6), respectively. This is shown in Figure 3. These figures and changes are similar to those detected in the whole study population.

We collected data on classical atherosclerosis risk factors, such as hypertension, diabetes mellitus, and hyperlipidemia, as well as patient-specific factors, including recipient age at HTX and CMV status. No patient-related characteristics were predictive of our measured CFR, IMR, and FFR values in a model that included these as predictors in addition to time. This is shown in Table 2.

## 4. Discussion

CAV remains a major limitation of the long-term clinical benefit of HTX. It has been typically described as diffuse and concentric narrowing of large epicardial and small intramyocardial arteries. The characteristic findings are intimal fibromuscular hyperplasia, atherosclerosis, and vasculitis. There are numerous similarities and differences in CAV with focal atherosclerotic lesions of proximal coronary arteries in native hearts. Intimal thickening and fibrofatty plaques are common to both, though fatty streaks, intimal erosions, fibrous cap thinning, hematomas, thrombosis, marked disruption of elastic laminae, and calcium deposition are less common in CAV [10].

The traditional morphological approach to diagnosing CAV has lately been complemented by functional diagnostic modalities. Reduced myocardial flow reserve in heart transplant patients demonstrated by positron emission tomography (PET) and cardiac magnetic resonance imaging (MRI) studies, consistent with vasculopathy, has been shown to be linked with decreased survival [11]. However, coronary functional studies performed in HTX recipients remain scarce.

The salient findings of our study are as follows: in patients with normal post-HTX invasive coronary angiograms receiving appropriate medical therapy, the functional status of the epicardial coronary arteries of transplanted hearts did not deteriorate over time, as evidenced by FFR remaining unchanged and highly above the ischemic threshold. On the contrary, a significant decrease in CFR was noted. In view of the increased IMR, this is caused by a significant deterioration in the function of the microvasculature. This seems especially the case after the first 12 months, when changes in IMR were also statistically significant. While the sequelae of epicardial disease may be fought by statins, aspirin, and revascularization [19], the best approach to microvascular disease is yet to be defined.

During the first year after HTX, Fearon et al. noted a significant decrease in IMR and FFR, suggesting that microvascular damage caused by the very transplantation may disappear by the end of the first year [12]. In the PITA II study, FFR decreased significantly from 0.90 early after HTX to 0.85 at the end of the first post-HTX year. This was found to be due to an increase in plaque volume (as measured by IVUS), a decrease in vessel volume, and a significantly improved IMR. IMR was shown to significantly decrease, a change driven mostly by those with very high post-HTX IMR values. CFR was found not to change significantly, likely because of the discordant changes in the epicardial and microvascular compartments.

Our observations complement this, since 90% of our measurements were performed after the first year. Our data suggest that after one year, the function of the microvasculature worsens, resulting in an increase in IMR and decrease in CFR even among those who present with angiographically normal-appearing coronary arteries with FFR values highly above 0.80. Our data should not be interpreted to prove that the physiology of the epicardial compartment does not deteriorate in the long run because we studied a non-random sample, and those with overt coronary artery stenoses at first examination were excluded, but microvascular function seems to worsen even among those who have ‘preserved’ epicardial physiology and normal-looking vessels. This should be viewed in the context that in the PITA study, 15% of the measured FFR values in asymptomatic patients with angiographically normal coronary arteries were <0.80 [20], highlighting the inability of invasive coronary angiography to reliably rule out CAV.

In 2016, after following 112 HTX patients for 4.5 years, Yang et al. found that patients with an IMR below 20 measured at 1 year after heart transplantation had a significantly better clinical outcome in terms of survival free of retransplantation compared with those with an IMR above 20 [21]. These authors also found that an FFR < 0.90 at baseline (soon after HTX) and rejection during the first year predicted lower cumulative event-free survival. Of note, an increase from the baseline to one-year IMR value was associated with a worse outcome, whereas IVUS-derived parameters were not independent predictors of death or retransplantation. But even in this analysis, no further physiology measurements were undertaken, so the long-term changes in epicardial and microvascular physiology were not captured.

Ahn et al. enrolled 237 patients who underwent an IMR measurement early after HTX. In their multicenter study, IMR measured early after heart transplantation was found to be associated with subsequent allograft rejection at 1 year and clinical events at 10 years [22]. IMR was found to be a stronger predictor of acute allograft rejection and long-term untoward events than clinical characteristics alone. These observations highlight the capability of microvascular functional measurements to predict future adverse events.

IMR measures the minimal microcirculatory resistance and as such is specific to microvasculature, as opposed to CFR which is influenced by the epicardial compartment as well. IMR has been shown to have a better reproducibility and lower coefficient of variation compared with CFR and was shown to be independent of hemodynamic conditions, as opposed to CFR [23]. CFR is a ratio that is dependent on baseline coronary blood flow, which shows significant variability, making it challenging to interpret CFR as it pertains to microvascular function. Presently, there are two methods to measure CFR: thermodilution and Doppler-based methods. The latter is based on measuring the average peak velocities of red blood cells at baseline and at peak hyperemia. Correlation between the two is modest, with thermodilution-based CFR being generally higher than Doppler-based CFR, with a mean difference of 0.6 [24]. In the present analysis, only thermodilution-based CFR was used. Lately, a new index, microcirculatory resistance reserve (MRR), was proposed to be the most specific to the microcirculation [25], but no study of MRR in HTX recipients has been published so far. On the contrary, wire-free, angiography-based IMR [26] and non-invasive studies [27] underline the importance of microvascular cardiac allograft vasculopathy in determining the long-term prognosis of HTX.

While our study was not powered to find significant correlation between FFR, CFR, IMR values and clinical outcomes, we were able to demonstrate changes in coronary physiology long-term after HTX.

Therapeutic interventions targeting both the immune and non-immune pathogenic mechanisms involved in CAV have been investigated with variable success. Non-immunological causes of CAV include old age, male gender, infections, dyslipidemia, diabetes, trauma during surgery, and reperfusion injury. Acute cellular rejection (ACR) occurring in the first 6–12 months after transplantation is an independent risk factor for CAV progression, with recurrent episodes having a cumulative effect on the onset of CAV. There is clearly a complex interplay between immunological and non-immunological risk factors, ultimately leading to endothelial injury and the development of disease [9]. Current clinical treatment for CAV is primarily focused on preventative strategies, including CMV infection prevention, rejection avoidance, vascular risk factor management, and specific pharmacotherapies, such as statins and mammalian target of rapamycin (mTOR) inhibitors that reduce the development of disease. Percutaneous revascularization for focal obstructive coronary stenosis and retransplantation for allograft dysfunction have also been increasingly utilized. These multi-faceted interventions have improved outcomes for patients with CAV over time [28], but CAV still remains a significant clinical entity to fight in the long run after HTX.

There are several limitations of our study. Due to the low number of patients, our statistical power to demonstrate a correlation of coronary physiology parameters and clinical events was limited. Furthermore, the measurements were not performed based on a regular schedule and were not consistently measured multiple times in every patient. The time between HTX and the first performed measurements show relatively big differences. The few patients who underwent coronary revascularization for focal epicardial stenoses were not included; however, we intended to study those without overt post-HTX coronary artery disease, and the remaining subjects were not randomly sampled in the present analysis. All the measurements were performed in the LAD since, in most subjects, this vessel supplies most myocardial mass, and its length makes thermodilution-based measurements feasible. However, investigating the same vessel in all patients made our study “population” more homogeneous.

## 5. Conclusions

Since CAV is a leading cause of death after HTX, prevention strategies must be implemented, and surveillance techniques targeting detection of the disease are essential. Invasive functional tests, such as FFR, CFR, and IMR, performed during surveillance coronary angiography offer the possibility of tracking the changes in coronary macro- and microvasculature, and thereby the early detection of CAV. Large-scale outcome trials are needed to define the clinical value of systematic invasive functional coronary artery testing in the long-term management of HTX patients.

## Figures and Tables

**Table 1:** Characteristics of the patients.

Patient Data	ALL Patients (N = 27)
Donor age; years ± SD	42 ± 8
Recipient age at HTX; years ± SD	54 ± 12
Recipient sex, male; n (%)	19 (70%)
Hypertension; n (%)	15 (56%)
Diabetes mellitus; n (%)	12 (44%)
Hyperlipidemia; n (%)	20 (74%)
Aspirin therapy; n (%)	19 (70%)
Statin therapy; n (%)	20 (74%)
BMI at first measurement; (kg/m^2^) ± SD	26 ± 4
LVEF; % ± SD	64 ± 10.8
TAPSE; mm ± SD	18 ± 3.4
LVOT VTI; mm ± SD	17 ± 9
RVSP; mmHg ± SD	40 ± 17
Cold ischemic time; minutes ± SD	179 ± 64
HTX indication: ischemic heart failure; n (%)	14 (52%)
Time from HTX to first measurement; months ± SD	43 ± 43
CMV-PCR positivity; n (%)	6 (23%)

BMI—body mass index; CMV-PCR—cytomegalovirus polymerase chain reaction; HTX—heart transplantation; LVEF—left ventricular ejection fraction; LVOT VTI—left ventricular outflow tract velocity time integral; RVSP—right ventricular systolic pressure; SD—standard deviation; TAPSE—tricuspid annular plane systolic excursion.

**Table 2:** Correlation of coronary physiology parameters and patient-related factors.

Outcome	Variable	Estimate	*p*-Value	Lower Limit of 95% CI	Upper Limit of 95% CI
CFR	CMV	−0.179	0.807	−1.731	1.373
CFR	DM	0.371	0.559	−0.989	1.731
CFR	HLP	−0.162	0.818	−1.738	1.414
CFR	HT	0.156	0.316	−0.789	2.220
CFR	age at HTX	−0.046	0.117	−0.105	0.013
IMR	CMV	−6.354	0.276	−18.41	5.697
IMR	DM	−5.691	0.259	−16.22	4.839
IMR	HLP	4.555	0.412	−7.631	16.74
IMR	HT	1.520	0.780	−10.23	13.26
IMR	age at HTX	−0.045	0.836	−0.508	0.418
FFR	CMV	−0.013	0.453	−0.048	0.022
FFR	DM	−0.017	0.272	−0.047	0.014
FFR	HLP	−0.019	0.260	−0.054	0.016
FFR	HT	0.017	0.300	−0.017	0.050
FFR	age at HTX	0.001	0.208	−0.001	0.002

CFR—coronary flow reserve, CI—confidence interval, CMV—cytomegalovirus, DM—diabetes mellitus, FFR—fractional flow reserve, HLP—hyperlipidemia, HT—hypertension, HTX—heart transplantation, IMR—index of microcirculatory resistance.

## Data Availability

The data that support the findings of this study are available from the corresponding author upon reasonable request.

## Abbreviations

ACEI	ACE inhibitor
ACR	acute cellular rejection
ARDS	acute respiratory distress syndrome
ASA	aspirin
CA	calcium channel blocker
CAV	cardiac allograft vasculopathy
CFR	coronary flow reserve
CI	confidence interval
CMV	cytomegalovirus
CMV-PCR	cytomegalovirus polymerase chain reaction
COVID-19	coronavirus disease 19
DSA	Donor-specific alloantibody
FFR	fractional flow reserve
HLA	human leukocyte antigen
HTX	heart transplantation
IMR	index of microcirculatory resistance
ISHLT	International Society of Heart and Lung Transplantation
IVUS	intravascular ultrasound
LAD	left anterior descending coronary artery
LVEF	left ventricular ejection fraction
LVOT VTI	left ventricular outflow tract velocity time integral
MRI	magnetic resonance imaging
mTOR	mammalian target of rapamycin
Pa	aortic pressure
Pd	distal (coronary) pressure
PET	positron emission tomography
PITA II	Physiologic Investigation for Transplant Arteriopathy II
RVSP	right ventricular systolic pressure
SD	standard deviation
TAPSE	tricuspid annular plane systolic excursion
T_mn,hyper_	hyperemic mean transit time
T_mn,rest_	resting mean transit time

## References

[1] Khush K.K., Cherikh W.S., Chambers D.C., Harhay M.O., Hayes D., Hsich E., Meiser B., Potena L., Robinson A., Rossano J.W. (2019). The International Thoracic Organ Transplant Registry of the International Society for Heart and Lung Transplantation: Thirty-sixth adult heart transplantation report—2019; focus theme: Donor and recipient size match. J. Heart Lung Transplant..

[2] Chih S., Chong A.Y., Mielniczuk L.M., Bhatt D.L., Beanlands R.S. (2016). Allograft Vasculopathy: The Achilles’ Heel of Heart Transplantation. J. Am. Coll. Cardiol..

[3] Sieg A., Weeks P., Krustchinsky L., Rajapreyar I. (2016). Statin therapy in cardiac allograft vasculopathy progression in heart transplant patients: Does potency matter?. Transplant. Rev..

[4] Mehra M.R., Crespo-Leiro M.G., Dipchand A., Ensminger S.M., Hiemann N.E., Kobashigawa J.A., Madsen J., Parameshwar J., Starling R.C., Uber P.A. (2010). International Society for Heart and Lung Transplantation working formulation of a standardized nomenclature for cardiac allograft vasculopathy-2010. J. Heart Lung Transplant. Off. Publ. Int. Soc. Heart Transplant..

[5] Fateh-Moghadam S., Bocksch W., Wessely R., Jäger G., Hetzer R., Gawaz M. (2003). Cytomegalovirus infection status predicts progression of heart-transplant vasculopathy. Transplantation.

[6] Delgado J.F., Reyne A.G., de Dios S., López-Medrano F., Jurado A., Juan R.S., Ruiz-Cano M.J., Dolores Folgueira M., Gómez-Sánchez M.Á., Aguado J.M. (2015). Influence of cytomegalovirus infection in the development of cardiac allograft vasculopathy after heart transplantation. J. Heart Lung Transplant. Off. Publ. Int. Soc. Heart Transplant..

[7] Topilsky Y., Gandhi M.J., Hasin T., Voit L.L., Raichlin E., Boilson B.A., Schirger J.A., Edwards B.S., Clavell A.L., Rodeheffer R.J. (2013). Donor-specific antibodies to class II antigens are associated with accelerated cardiac allograft vasculopathy: A three-dimensional volumetric intravascular ultrasound study. Transplantation.

[8] Loupy A., Toquet C., Rouvier P., Beuscart T., Bories M.C., Varnous S., Guillemain R., Pattier S., Suberbielle C., Leprince P. (2016). Late failing heart allografts: Pathology of cardiac allograft vasculopathy and association with antibody-mediated rejection. Am. J. Transplant..

[9] Raichlin E., Edwards B.S., Kremers W.K., Clavell A.L., Rodeheffer R.J., Frantz R.P., Pereira N.L., Daly R.C., McGregor C.G., Lerman A. (2009). Acute cellular rejection and the subsequent development of allograft vasculopathy after cardiac transplantation. J. Heart Lung Transplant..

[10] Rahmani M., Cruz R.P., Granville D.J., McManus B.M. (2006). Allograft vasculopathy versus atherosclerosis. Circ. Res..

[11] Konerman M.C., Lazarus J.J., Weinberg R.L., Shah R.V., Ghannam M., Hummel S.L., Corbett J.R., Ficaro E.P., Aaronson K.D., Colvin M.M. (2018). Reduced myocardial flow reserve by positron emission tomography predicts cardiovascular events after cardiac transplantation. Circ. Heart Fail..

[12] Fearon W.F., Hirohata A., Nakamura M., Luikart H., Lee D.P., Vagelos R.H., Hunt S.A., Valantine H.A., Fitzgerald P.J., Yock P.G. (2006). Discordant Changes in Epicardial and Microvascular Coronary Physiology after Cardiac Transplantation: Physiologic Investigation for Transplant Arteriopathy II (PITA II) Study. J. Heart Lung Transplant..

[13] Pijls N.H., De Bruyne B., Smith L., Aarnoudse W., Barbato E., Bartunek J., Bech G.J., Van De Vosse F. (2002). Coronary thermodilution to assess flow reserve: Validation in humans. Circulation.

[14] Candreva A., Gallinoro E., van ‘t Veer M., Sonck J., Collet C., Di Gioia G., Kodeboina M., Mizukami T., Nagumo S., Keulards D. (2021). Basics of Coronary Thermodilution. JACC Cardiovasc. Interv..

[15] Escaned J., Flores A., García-Pavía P., Segovia J., Jimenez J., Aragoncillo P., Salas C., Alfonso F., Hernández R., Angiolillo D.J. (2009). Assessment of microcirculatory remodeling with intracoronary flow velocity and pressure measurements: Validation with endomyocardial sampling in cardiac allografts. Circulation.

[16] Fearon W.F., Balsam L.B., Farouque H.M., Caffarelli A.D., Robbins R.C., Fitzgerald P.J., Yock P.G., Yeung A.C. (2003). Novel index for invasively assessing the coronary microcirculation. Circulation.

[17] Pinheiro J., Bates D. (2006). Mixed-Effects Models in S and S-PLUS.

[18] Kuznetsova A., Brockhoff P.B., Christensen R.H.B. (2017). lmerTest Package: Tests in Linear Mixed Effects Models. J. Stat. Softw..

[19] Orban M., Kuehl A., Pechmajou L., Mueller C., Hausleiter J., Sfeir M., Bories M.C., Martin A.C., Ulrich S.M., Dalla Pozza R. (2023). Reduction of cardiac allograft vasculopathy by PCI: Quantification and correlation with outcome after heart transplantation. Eur. Heart J..

[20] Fearon W.F., Nakamura M., Lee D.P., Rezaee M., Vagelos R.H., Hunt S.A., Fitzgerald P.J., Yock P.G., Yeung A.C. (2003). Simultaneous assessment of fractional and coronary flow reserves in cardiac transplant recipients: Physiologic Investigation for Transplant Arteriopathy (PITA study). Circulation.

[21] Yang H.M., Khush K., Luikart H., Okada K., Lim H.S., Kobayashi Y., Honda Y., Yeung A.C., Valantine H., Fearon W.F. (2016). Invasive Assessment of Coronary Physiology Predicts Late Mortality after Heart Transplantation. Circulation.

[22] Ahn J.M., Zimmermann F.M., Gullestad L., Angerås O., Karason K., Russell K., Lunde K., Okada K., Luikart H., Khush K.K. (2021). Microcirculatory Resistance Predicts Allograft Rejection and Cardiac Events after Heart Transplantation. J. Am. Coll. Cardiol..

[23] Ng M.K.C., Yeung A.C., Fearon W.F. (2006). Invasive Assessment of the Coronary Microcirculation. Superior Reproducibility and Less Hemodynamic Dependence of Index of Microcirculatory Resistance Compared with Coronary Flow Reserve. Circulation.

[24] Demir O.M., Boerhout C.K.M., de Waard G.A., van de Hoef T.P., Patel N., Beijk M.A.M., Williams R., Rahman H., Everaars H., Kharbanda R.K. (2022). Comparison of Doppler Flow Velocity and Thermodilution Derived Indexes of Coronary Physiology. J. Am. Coll. Cardiol. Interv..

[25] De Bruyne B., Pijls N.H.J., Gallinoro E., Candreva A., Fournier S., Keulards D.C.J., Sonck J., van’t Veer M., Barbato E., Bartunek J. (2021). Microvascular Resistance Reserve for Assessment of Coronary Microvascular Function. J. Am. Coll. Cardiol..

[26] Weiss K.J., Hohendanner F., Diedrichs F., Reiber J.H.C., Castillo Tovar J., Falk V., Schoenrath F., Stawowy P., Just I.A. (2023). Angio-IMR identifies microvascular cardiac allograft vasculopathy in heart transplant recipients. Eur. Heart J..

[27] Clerkin K.J., Topkara V.K., Farr M.A., Jain R., Colombo P.C., Restaino S., Sayer G., Castillo M., Lam E.Y., Chernovolenko M. (2022). Noninvasive physiologic assessment of cardiac allograft vasculopathy is prognostic for post-transplant events. J. Am. Coll. Cardiol..

[28] Tremblay-Gravel M., Racine N., de Denus S., Ducharme A., Pelletier G.B., Giraldeau G., Liszkowski M., Parent M.C., Carrier M., Fortier A. (2017). Changes in outcomes of cardiac allograft vasculopathy over 30 years following heart transplantation. JACC Heart Fail..

